# Gut Microbiome Is Related to Cognitive Impairment in Peritoneal Dialysis Patients

**DOI:** 10.3390/nu16162659

**Published:** 2024-08-12

**Authors:** Fabiola Martín-del-Campo, Natali Vega-Magaña, Noé A. Salazar-Félix, Alfonso M. Cueto-Manzano, Marcela Peña-Rodríguez, Laura Cortés-Sanabria, María L. Romo-Flores, Enrique Rojas-Campos

**Affiliations:** 1 Biomedical Research Unit 02, Specialties Hospital, Western National Medical Center, Mexican Institute of Social Security, Belisario Dominguez #1000, Guadalajara 44320, Mexico; fabi_mc@hotmail.com (F.M.-d.-C.); alejandrosalazarfelix@gmail.com (N.A.S.-F.); cortes_sanabria@yahoo.com.mx (L.C.-S.); erojascampos@yahoo.com.mx (E.R.-C.); 2 Laboratory of Pathology, Department of Microbiology and Pathology, Health Sciences University Center, University of Guadalajara, Sierra Mojada #950, Guadalajara 44350, Mexico; natalivega.dcb@gmail.com; 3 Research Institute on Chronic and Degenerative Diseases, Department of Molecular Biology and Genomics, Health Sciences University Center, University of Guadalajara, Sierra Mojada #950, Guadalajara 44350, Mexico; marcela.pena@cucs.udg.mx; 4 Department of Nephrology, Regional General Hospital 46, Mexican Institute of Social Security, Lázaro Cárdenas Av. 1060, Guadalajara 44910, Mexico; mlromof@hotmail.com

**Keywords:** gut microbiota, cognitive impairment, peritoneal dialysis

## Abstract

Gut microbiota disturbances may influence cognitive function, increasing uremic toxins and inflammation in dialysis patients; therefore, we aimed to evaluate the association of the gut microbiota profile with cognitive impairment (CI) in patients on automated peritoneal dialysis (APD). In a cross-sectional study, cognitive function was evaluated using the Montreal Cognitive Assessment in 39 APD patients and classified as normal cognitive function and CI. The gut microbiota was analyzed using the 16S rRNA gene sequencing approach. All patients had clinical, biochemical and urea clearance evaluations. Eighty-two percent of patients were men, with a mean age of 47 ± 24 years and 11 (7–48) months on PD therapy; 64% had mild CI. Patients with CI were older (53 ± 16 vs. 38 ± 14, *p* = 0.006) and had a higher frequency of diabetes mellitus (56% vs. 21%, *p* = 0.04) and constipation (7% vs. 48%, *p* = 0.04) and lower creatinine concentrations (11.3 ± 3.7 vs. 14.9 ± 5.4, *p* = 0.02) compared to normal cognitive function patients. Patients with CI showed a preponderance of S24_7, Rikenellaceae, Odoribacteraceae, *Odoribacter* and *Anaerotruncus*, while patients without CI had a greater abundance of *Dorea*, *Ruminococcus*, *Sutterella* and *Fusobacteria* (LDA score (Log_10_) > 2.5; *p* < 0.05). After glucose and age adjustment, Odoribacter was still associated with CI. In conclusion, patients with CI had a different gut microbiota characterized by the higher abundance of indole-producing and mucin-fermenting bacteria compared to normal cognitive function patients.

## 1. Introduction

Cognitive function declines as chronic kidney disease progresses [1]; a cognitive impairment (CI) prevalence of 49.1% in hemodialysis [2] and 28.7% in peritoneal dialysis (PD) [3] has been reported. In a recent study, CI was reported in 65% of PD patients in our setting [4]. This condition may limit patient self-care and treatment adherence [5], increasing the mortality risk [6]. Neurocognitive deterioration in patients with chronic kidney disease is proposed to be the result of traditional cardiovascular risk factors (older age, hypertension, diabetes) combined with vascular (uremic toxins, inflammation, dialysis-related hemodynamic changes) and non-vascular kidney-related factors (oxidative stress, anemia, malnutrition) [7].

On the other hand, the gut microbiome may influence the brain and behavior by means of immune, endocrine and neural pathways, thus increasing the risk of neuropsychiatric disorders [8]. Chronic kidney disease patients have evident alterations in the gut microbiota composition compared to the healthy population [9,10], characterized by bacterial overgrowth, an increase in proteolytic/saccharolytic fermentation, intestinal epithelial disruption and bacterial translocation [11]. Gut dysbiosis is associated with higher uremic toxin production and inflammation [12], as well as with a decrease in short-chain fatty acid production [10]. Patients treated with PD show a different microbiota composition (associated with negative outcomes) compared with non-dialysis and hemodialysis patients, probably related to their long-term dextrose dialysate exposure [13]. However, the association of the gut microbiota with CI in patients with chronic kidney disease has been poorly studied [14,15], particularly in PD patients. Therefore, the aim of the present study was to evaluate the association of the gut microbiota profile with the cognitive function of patients on automated PD.

## 2. Methods

In a cross-sectional study, patients attending the PD unit in a secondary-care hospital, older than 18 years and with an APD treatment duration longer than 3 months, were invited to participate in this study. Patients with illiteracy; those on anti-inflammatory drugs or antibiotics; and those with a visual disability, neurodegenerative or psychiatric disorder, infectious disease (6 weeks prior), cancer, AIDS, heart or liver failure, inflammatory bowel disease or severe intestinal malabsorption were excluded.

Written informed consent was obtained from all patients, and they subsequently had a clinical, biochemical and cognitive evaluation. Biochemical tests were performed after 10 h of fasting by habitual techniques and included a blood cell count, serum chemistry, lipid profile and electrolytes. C-reactive protein was measured by immunoturbidimetry. Serum lipopolysaccharides were measured by double-antibody sandwich ELISA (MyBiosource, San Diego, CA, USA). Residual kidney function was calculated from urea nitrogen and creatinine [16], and the total urea clearance (Kt/Vurea) was reported (urine + dialysate). The protein nitrogen appearance was calculated and normalized by the ideal body weight (nPNA) [17]. PD was performed according to the treating nephrologist’s individual prescription, on a Homechoice Cycler™ (Baxter, Deerfield, IL, USA) using standard dextrose-based dialysis fluid (Baxter, Cuernavaca, Mexico). The nutritional evaluation consisted of body mass index calculation, the mid-arm muscle area, a subjective global assessment and a dietary intake evaluation (24 h dietary recall). Ethical approval was obtained from the Local Research and Ethics Committee 1301 with the registration number R-2020-1301-176 (22 October 2020).

***Cognitive function evaluation***. Patients were classified according to cognitive function using the Montreal Cognitive Assessment (MoCA), which provides a continuous score from 0 to 30 points, evaluating orientation, delayed recall, visuospatial/executive capabilities, language, naming, attention and abstraction; a score ≤25 was considered as indicative of CI. If a patient’s schooling had lasted less than 12 years, one point was added to reduce the educational bias [18].

***Stool sample collection***. Patients were instructed to collect 1–2 g stool samples using sterile bottles and wooden sticks; the samples were refrigerated from collection to delivery at the hospital and then immediately transferred into Eppendorf tubes within the first 12–24 h of collection; the samples were stored at −80 °C for further microbiota evaluation. The stool sample was collected on the same day as the cognitive function evaluation.

***16S rRNA sequencing***. The Quick-DNA Fecal/Soil Microbe Miniprep Kit (Zymo Research, Irvine, CA, USA) was used for DNA extraction. Subsequently, the V3-V4 region of the 16S rRNA gene was amplified and sequenced with the Illumina technology using the MiSeq platform (MiSeq reagent Kit V3, 600-cycle Illumina, Albany, NY, USA). The primers employed were as follows.

F: 5′TCGTCGGCAGCGTCAGATGTGTATAAGAGACAGCCTACGGGNGGCWGCAG-3′.

R: 5′GTCTCGTGGGCTCGGAGATGTGTATAAGAGACAGGACTACHVGGGTATCTAATCC-3′.

Polymerase chain reactions were performed with the Platinum Taq DNA Polymerase High Fidelity (Thermo Fisher Scientific, Carlsbad, CA, USA). Amplicons were initially purified using magnetic beads (Agencourt AMPure XP, Beckman Coulter, Brea, CA, USA) and quantified with high-sensitivity kits (Qubit dsDNA high-sensitivity, Thermo Fisher Scientific, Carlsbad, CA, USA); then, spectrophotometry was used for purity verification and the size was verified by electrophoresis. For further sample identification, a combination of specific indexes for each subject was added (Nextera XT Index A Kit, Illumina, Albany, NY, USA). The library was purified and quantified and the amplicon size verified anew. Finally, the samples were placed in an equimolar solution and mixed with 20% PhiX (Illumina, Albany, NY, USA).

***Bioinformatic and statistical analysis***. The analysis of sequences was performed with the QIIME 2-2023.2 platform. Forward and reverse files were merged and filtered to remove low-quality sequences (Phred scale < Q30, forward 280 pb, reverse 240 pb cut-off). Subsequently, the sequences were clustered with >0.1% representation into Amplicon Sequence Variants (ASVs) for further analysis. Bacterial alpha diversity was calculated with the observed features, Chao 1 and Shannon indices, while beta diversity was determined with the Jaccard dissimilarity index and plotted using principal component analysis. Differential abundance analysis was performed with the linear discriminant analysis effect size (LEfSe) on the Galaxy and Microbiome Analyst platforms. Random forest analysis and the creation of a heatmap via abundance analysis were performed on the Microbiome Analyst platform. The functional profiles of microbial communities were analyzed in Rstudio v2023.12.1 using the Microeco package v1.8.0. Pearson correlations were also obtained with the Galaxy platform and observed in the Cytoscape software 3.7.2; a *p* value < 0.05 and r = ±0.5 were considered statistically significant.

Quantitative variables are shown as the mean ± standard deviation or median (25–75% percentiles) according to the data distribution, and qualitative ones are shown as frequencies (percentage). Comparisons between mild CI and normal cognitive function patients were performed with the Mann–Whitney U, χ^2^ or Fisher exact test as appropriate, using SPSS v.23. A *p* value < 0.05 was considered statistically significant.

## 3. Results

Thirty-nine APD patients were included in the study; 82% were men and the mean age was 47 ± 17 years. CI was present in 25 (64%) patients, all cases with a mild grade. The socio-demographic and clinical characteristics according to the presence of CI are shown in Table 1. Patients with mild CI were significantly older, had diabetes mellitus and constipation more frequently and exhibited a non-significant trend towards a longer time on PD therapy compared with patients with normal cognitive function. No other differences were found.

Patients with mild CI also had significantly lower serum creatinine than normal cognitive function patients; other biochemical or dialysis-related variables were not different between groups (Table 2).

**Figure 1:**
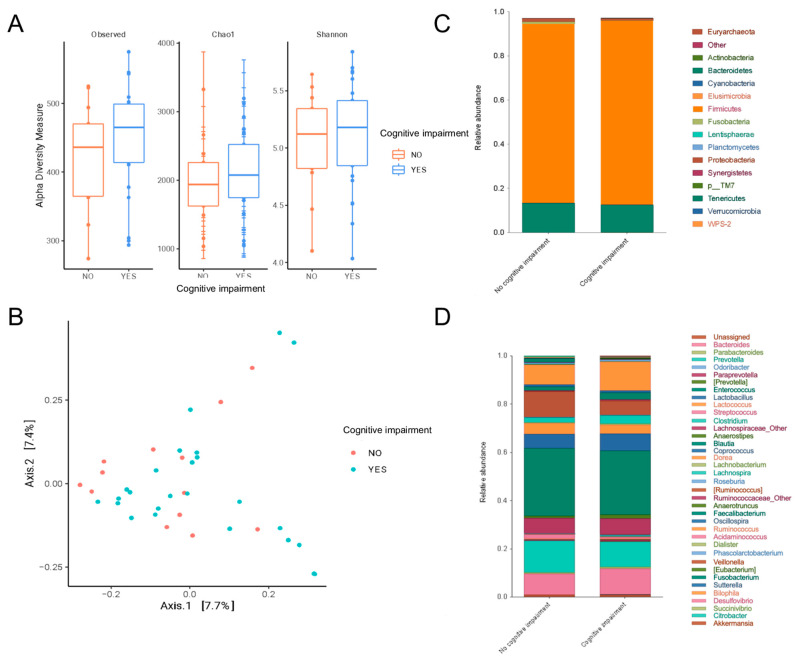
Microbiome composition analysis in patients with peritoneal dialysis and cognitive impairment. (**A**) Alpha diversity measured by observed features, Chao1 and Shannon indices. (**B**) Beta diversity analysis by Jaccard method. (**C**) Phylum relative abundance. (**D**) Genus relative abundance. Statistical analysis was performed with Wilcoxon and ANOSIM.

The composition of the gut bacteria according to the presence of mild cognitive impairment is shown in Figure 1. The alpha diversity indices (observed features, Chao1 and Shannon) indicate higher gut microbiome diversity in patients with mild CI; nevertheless, there was no significant difference between the groups (Figure 1A). Similarly, the beta diversity analysis did not show significant differences (Figure 1B). Regarding the relative abundance, it was observed that Firmicutes was the predominant phylum in both groups. The Fusobacteria phylum had a higher presence in patients without CI (Figure 1C) than in patients with CI. On the other hand, the abundance of *Bacteroides*, *Anaerostipes*, *Coprococcus*, *Faecalibacterium* and *Lachnospira* was higher in patients with CI. In contrast, *Prevotella*, *Dorea* and *Fusobacterium* were overrepresented in patients without CI (Figure 1D).

**Figure 2:**
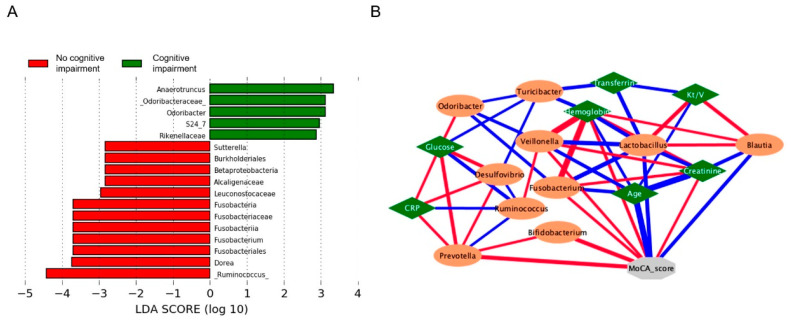
Differential abundance analysis and correlations in patients with peritoneal dialysis and cognitive impairment. (**A**) LEfSe analysis showed that cognitive impairment was characterized by *Anaerotruncus* and *Odoribacter*. whereas *Ruminococcus*, *Dorea*, *Fusobacterium* and *Sutterella* represented patients without cognitive impairment. (**B**) Pearson correlations showed that the MoCA score had a positive correlation with *Fusobacterium*, which in turn had a negative correlation with *Odoribacter*. On the other hand, *Odoribacter* had a positive correlation with glucose levels, which, at the same time, had a positive correlation with C-reactive protein levels. A positive correlation is represented by red lines and blue lines indicate negative ones. *p* < 0.05 and r = ±0.5 were considered significant.

Furthermore, the differential abundance analysis showed that the CI group was enriched in *Odoribacter*, *Anaerotruncus*, *S24_7* and Rikenellaceae. In contrast, *Ruminococcus*, *Dorea*, *Fusobacterium* and *Sutterella* characterized patients without CI (Figure 2A). Moreover, the Pearson correlation analysis exhibited a positive correlation between the MoCA score and *Fusobacterium*, which, in turn, had a negative correlation with *Odoribacter*. Meanwhile, *Odoribacter* had a positive correlation with the glucose level, which, at the same time, had a positive correlation with the CRP level (Figure 2B).

**Figure 3:**
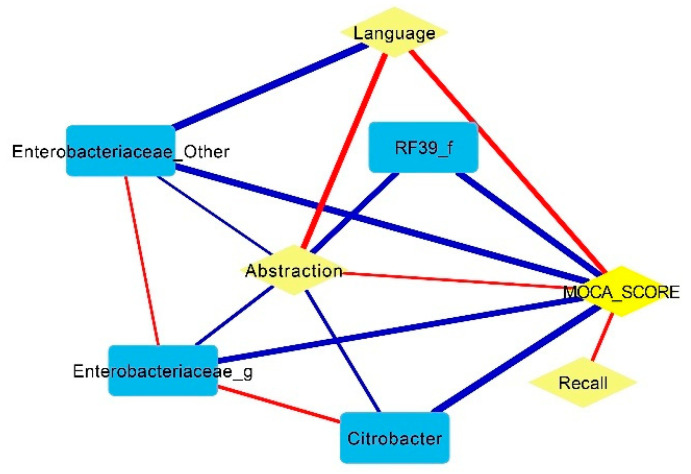
Pearson correlation network linkages of MoCA domains in CI patients. A positive correlation is represented by red lines and blue lines indicate negative ones. *p* < 0.05 and r = ±0.5 were considered significant.

In addition, the Pearson correlation network linkages in CI patients (Figure 3) showed a negative association of *Enterobacteriaceae* with MoCA (r −0.62, *p* = 0.006), abstraction (r −0.69, *p* = 0.006) and language (r −0.60, *p* < 0.0001). Meanwhile, *Citrobacter* had a strong positive correlation with *Enterobacteriaceae* (r 0.71, *p* = 0.006) and a negative correlation with abstraction (r −0.70, *p* < 0.0001) and the MoCA score (r −0.60, *p* < 0.0001). The abstraction, language and recall domains were the main contributors to the MoCA score.

**Figure 4:**
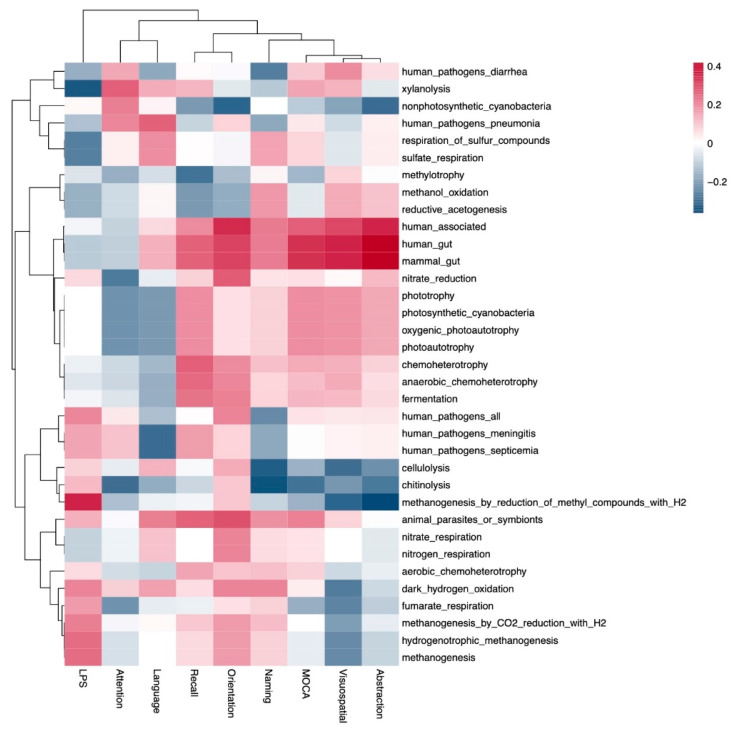
Functional profiles of microbial communities and correlation network linkages. The heatmap represents the Spearman correlations between the functional profiles of the microbial communities, MoCA domains, MoCA score, and LPS levels.

Subsequently, we obtained the functional profile via microbial community analysis, which was represented as a heatmap of Spearman correlations with clinical variables of interest, such as the domains of MoCA (abstraction, visuospatial, naming, orientation, recall, language and attention), the MoCA score and the LPS levels (Figure 4). No significant differences were observed in this analysis; however, a non-significant trend for methanogenesis_by_reduction_of methyl_compounds_with_H2 metabolism was associated with lower abstraction and higher serum lipopolysaccharides; the MoCA score, as well as its domains had a positive correlation with human_associated, human_gut and mammal_gut and a negative correlation with cellulolysis and chitinolysis.

**Figure 5:**
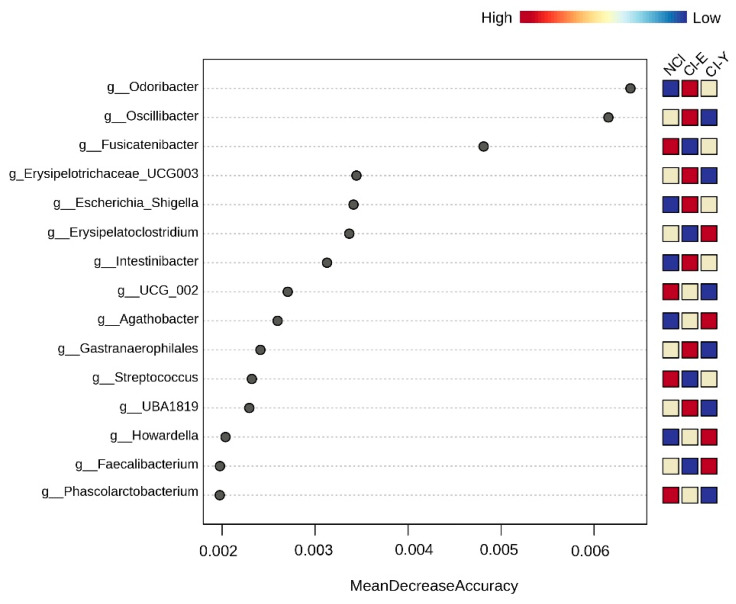
Random forest analysis. Variable importance plot of random forest analysis excluding the glucose level variable. The variables are shown in descending order of importance (according to the mean decrease accuracy value shown); a higher value for the mean decrease in accuracy reflects the higher importance of the variable in the model. NCI: no cognitive impairment; CI-E: cognitive impairment in elder patients; CI-Y: cognitive impairment in younger patients.

On the other hand, a random forest analysis (Figure 5) was used with the glucose levels as a covariate. We further categorized the patients by age range as CI-E (>60 years old) and CI-Y (<60 years old). This analysis revealed a distinct microbiome signature in CI-E patients, characterized by *Odoribacter*, *Oscillibacter*, *Erysipelotrichaceae_UCG_003*, *Escherichia_Shigella*, *Intestinibacter*, *Gastranaerophilales* and *UBA_1819*. In contrast, *Erysipelatoclostridium*, *Agathobacter*, *Howardella* and *Faecalibacterium* were prominent in CI-Y patients.

## 4. Discussion

Gut microbiota disturbances have been clearly identified in PD patients, with differences related to the dialysis vintage, dextrose exposure and the presence of residual kidney function [19] and associated with clinical outcomes, such as PD-associated peritonitis [13] and vascular calcification [20]. However, the association between the gut microbiota composition and cognitive function in patients on PD has been poorly studied.

In hemodialysis patients, SCFA-producing bacteria such as *Faecalibacterium* and *Bifidobacterium* have shown a positive correlation with different cognitive function domains [21]; additionally, patients with cognitive impairment had higher concentrations of gut-derived metabolites, such as the polyamine putrescine [14]. In PD patients, the gut microbiota was recently explored regarding cognitive function, and, in contrast to our results, *Prevotellaceae* was enriched in cognitive impairment patients; however, similarly to our study, other SCFA-producing bacteria were decreased in patients with CI [22].

CI was present in 64% of patients and was associated with older age, the presence of diabetes and lower serum creatinine concentrations, as previously shown by Salazar-Felix et al. [4]. The lower serum creatinine in patients with CI may have been related to their low muscle mass rather than the different dialysis doses, as the volume and Kt/V_urea_ were similar between the groups; moreover, patients with CI had a greater proportion of low muscle mass and slightly lower serum albumin than those without CI. Recently, it has been suggested that patients with >60 months on PD tend to exhibit an improvement in the gut microbiota and its metabolites [23]; in the present study, the patients had a shorter dialysis vintage (only three had a duration of more than 60 months) and those with CI had a slightly longer time on dialysis but without significant differences. Whether the modification or stabilization of the gut microbiota could be associated with CI in long-term PD deserves further analysis.

The alpha and beta diversity were not different among PD patients with and without CI. In previous studies, the alpha diversity has shown controversial results in neuropsychiatric disorders such as depression or Alzheimer’s disease spectrum (including CI); therefore, it has been considered as a poor indicator of the intestinal microbiota in this group of patients [24,25]. Regarding beta diversity, the results are also inconsistent in Alzheimer’s disease spectrum patients; including a comparison of the weighted and unweighted UniFrac distances, most of the studies showed no differences between patients with Alzheimer’s disease, mild CI and healthy controls [25].

Patients with CI had a greater abundance of members of the *Odoribacteraceae*, *Muribaculaceae* (previously known as S24_7) and *Rikenellaceae* families, as well as the *Anaerotruncus* and *Odoribacter* genera. *Muribaculaceae* and *Rikenellaceae* bacteria belong to the Bacteroidales class, along with Odoribacteraceae; however, unlike the latter, they are mucolytic bacteria that ferment host-derived glycans. In the human gut, the main mucin source is the gastrointestinal epithelium, and mucin fermentation allows bacterial survival and adhesion, as host-derived glycans are used as bacterial energy sources when the fermentable carbohydrate intake is reduced [26]. Mucin degradation by bacteria could be beneficial as it promotes mucosal glycan production, protecting the intestinal barrier. However, host-derived glycans also protect host cells from potential pathogenic bacteria, and mucolytic bacteria overgrowth may induce gut mucus layer damage, as well as the increased release of some enteric pathogens that use monosaccharides produced through mucin degradation [27]. Hence, the biological significance of this bacterial metabolism in kidney disease patients and CI deserves further research.

*Anaerotruncus* belongs to the Oscillospiraceae family and it mainly ferments carbohydrates; however, it is also indole-positive [28]. In patients with Parkinson’s disease, a higher abundance of *Odoribacter* and *Anaerotruncus* genera has been associated with CI, similar to our results [29,30]. Furthermore, *Anaerotruncus* has consistently been associated with CI [31] and the presence of motor and non-motor symptoms in patients with Parkinson’s [32], neuroinflammation and higher amyloid-B levels in Alzheimer’s disease experimental models [33]. Likewise, in patients with major depressive disorder, the *Odoribacter* abundance was reported to be higher compared to healthy controls; however, it was not associated with cognitive function [34]. In experimental models, aged mice presented a gut microbiota enriched in *Odoribacter* and *Porphyromonadaceae*, which was associated with anxiety-like behavior [35]. Interestingly, controversial data have been published, particularly in Alzheimer’s disease, regarding *Odoribacter*. In mice models, it has been associated with impaired spatial memory and B-amyloid plaque deposition [36]; meanwhile, in others, an increase in *Odoribacter* abundance improved short-term memory and cognitive abilities [37]. In humans, the presence of *Odoribacter* was positively associated not only with cognitive function but also with the brain structure, such as a larger right hippocampus volume and acetic acid concentrations, representing a protective factor against neurological damage in Alzheimer’s disease patients [38]. Bacteria belonging to the *Odoribacter* genus are predominantly found in the human gut, with beneficial effects in maintaining a healthy gut and preventing inflammation because of their ability to ferment carbohydrates and produce short-chain fatty acids [39]; however, they are also recognized as opportunistic pathogens and, as in the case of *Anaerotruncus*, are able to use amino acids as carbon sources, therefore producing indole from tryptophan [40]. Notably, indole metabolites can cross the blood–brain barrier, increasing neuroinflammation and apoptosis, and have been associated with CI in non-dialysis chronic kidney disease and hemodialysis patients [41,42].

On the other hand, patients without CI had a higher abundance of *Ruminococcus*, *Dorea* and *Sutterella*. *Ruminococcus*, along with *Dorea*, has previously been found to be decreased in neurological diseases such as Parkinson’s [30]. *Ruminococcus* belongs to the class Clostridia; it is able to ferment complex carbohydrates such as cellulose and starch in the colon and is associated with gastrointestinal health, probably related to short-chain fatty acid production, particularly acetate, which stimulates butyrate production by other butyrate-producing bacteria [43]. *Dorea* and *Sutterella* are also carbohydrate-fermenting bacteria, producing acetate and, to a lower extent, butyrate from pectins [44].

The *Fusobacterium* genus deserves special attention, as it was also more abundant in patients without CI and had a positive correlation with the MoCA score. *Fusobacterium* in the oral cavity has been studied in neurocognitive diseases, as it has been frequently found to be associated with CI and Alzheimer’s disease; distinctively, *Fusobacterium nucleatum* is a pathogen that grows in periodontitis [45]. In patients with chronic kidney disease, *Fusobacterium* is consistently increased, along with nitrogen-containing compound fermenters [46]. Protein fermentation produces uremic toxins (phenol, indole, ammonia), but also, in some cases, it is possible to obtain branched-chain amino acids as well as short-chain fatty acids. Interestingly, *Fusobacterium* species are able to produce butyrate via the glutamate degradation pathway, either via pyruvate or crotonyl-CoA [47]. Thus, *Fusobacterium*’s significance in the chronic kidney disease patient context deserves further investigation.

The inter-individual variation in the gut microbiota is mainly affected by the dietary carbohydrate intake. The gut microbiota of patients with normal cognitive function was characterized by the higher abundance of bacteria with plant structural polysaccharide degradation activity (*Ruminococcus*, *Dorea*, *Sutterella*), such as cellulose and resistant starch, commonly present in cereals, as well as pectins, commonly present in fruits. On the other hand, patients with CI were characterized by a gut microbiota enriched in indole producers (*Odoribacter*, *Anaerotruncus*), as well as mucin-fermenting bacteria (*Muribaculaceae*, *Rikenellaceae*). Patients with chronic kidney disease have greater intestinal availability of protein and amino acids in the gut, associated with the presence of uremia, malabsorption and protein energy wasting; in addition, these patients frequently have a very low-fiber diet, associated not only with anorexia but also with excessive potassium restriction, limiting their vegetable, fruit and legume intake [48], which in turn could be associated with the negative metabolism of the gut microbiota due to dysbiosis.

In the correlation network, some bacterial groups were found to be associated with the MoCA score, mainly a positive association with *Fusobacterium*, *Bifidobacterium* and *Prevotella* and a negative association with *Blautia*. *Prevotella* is one of the main genera positively associated with brain connectivity, in the recognition and attention areas, as well as cognitive function, including control over multitasking, episodic memory retrieval and visual or language information processing [49,50]. *Prevotella* was also associated with emotional well-being [50,51]. *Bifidobacterium* has been associated with attention- and memory-related brain networks, as its higher abundance contributed to the increased connectivity of the medial prefrontal cortex of the default mode network and parietal regions; moreover, along with *Prevotella*, it modulates the fronto-parietal attention network. On the other hand, the higher abundance of *Blautia* was associated with the stress-induced executive control network and default mode network connectivity [51]. The *Prevotella* and *Bifidobacterium* genera are well-recognized anti-inflammatory genera, consistently decreased in neurocognitive diseases. Anti-inflammatory bacteria produce chemical signaling (short-chain fatty acids) with positive effects on immune activation, thus regulating neuroinflammation. Additionally, *Bifidobacterium* may produce neuroactive substances such as GABA or neurotransmitters and amino acid precursors, hence modulating related diseases and behavioral changes [52].

Notably, *Odoribacter* was also positively related to the serum glucose concentrations in the current study. Recently, *Odoribacter* has been associated with lower insulin resistance in obese patients [53] and a lower type 2 diabetes mellitus risk [54]. It is possible that the presence of chronic kidney disease is associated with the negative metabolism of *Odoribacter* bacteria, as described above.

The gut microbiota composition is influenced by several factors, including age, genetics, lifestyle, geographical area and the presence of comorbidities. A meta-analysis to identify the gut microbiota characteristics associated with neurocognitive alterations in those on the Alzheimer’s spectrum, including mild CI, showed that the microbiota may change according to the CI severity but also according to geographical variations [25]. In the present study, although the results were consistent with the previous literature, some findings were contradictory, such as the positive association of *Fusobacterium* with better cognitive function and the negative role of *Odoribacter*; this could have been influenced by geographical differences and particularly the presence of chronic kidney disease. PD patients have shown a different gut microbiota compared with non-dialysis chronic kidney disease or hemodialysis patients [13]; these differences may be associated with the uremic gut environment, dietary restrictions and pharmacological interventions, but the different dialysis procedures may also exert an influence.

When the MoCA domains were considered in the correlation network, the *Enterobacteriaceae* family and *Citrobacter* genus were associated with lower abstraction and lower total MoCA scores, which could indicate a negative effect of pathogens/pathobionts (inflammation and toxin production). *Citrobacter* is a sulfur-reducing pathogenic bacterium associated with increased gut permeability, inflammatory bowel disease and neurotoxicity [55,56], as well as with constipation-associated peritonitis in PD patients [57]. In line with this, methanogenesis metabolism was associated with lower abstraction and higher serum lipopolysaccharides; the methanogenesis profile has been also associated with constipation, commonly present in neurodegenerative diseases such as Parkinson’s, increasing the intestinal permeability and exposure to gut neurotoxins [58,59]. Additionally, it has been observed that methanogenic bacteria may decrease the beneficial butyrate production in the gut [60].

Finally, when cognitive function was analyzed by age group, adjusted by serum glucose, *Odoribacter* was still associated with CI. Other mucolytic bacteria, such as *Intestinibacter* and *UBA1819*, were overrepresented in patients with CI, which, in turn, may promote pathogen/pathobiont adherence (*Escherichia-Shigella*, *Gastranaerophilales*) in the gut and increase inflammation and toxin exposure [27].

This study has limitations associated with its cross-sectional nature and the relatively small sample size; however, the novel results provide evidence of the possible relationship between the gut microbiota composition and the presence of CI in patients on PD. Whether dysbiosis is the cause or effect of CI in dialysis patients deserves further research, as well as the study of gut microbiota modification by means of nutritional or pharmacological interventions to improve the burden of CI in this group of patients.

## 5. Conclusions

CI was present in two thirds of these automated PD patients, who had a greater abundance of the *Odoribacter* genus compared with those patients with normal cognitive function. Better cognitive function, according to the MoCA score, was positively associated with SCFA-producing bacteria such as *Prevotella* and *Bifidobacterium*.

## Figures and Tables

**Table 1:** Comparison of sociodemographic and clinical results according with the presence of cognitive impairment.

Variable	Normal Cognitive Function (n = 14)	Mild Cognitive Impairment (n = 25)	*p*
Age (years)	38 ± 14	53 ± 16	0.006
Female sex, n (%)	2 (14)	5 (20)	0.66
Marital status, n (%)			0.60
Single	5 (36)	5 (20)	
Married	7 (50)	18 (72)	
Widowed/divorced	2 (14)	2 (8)	
Educational level, n (%)			0.49
Elementary/middle school	7 (50)	17 (68)	
High school/technical career	5 (36)	5 (20)	
Professional	2 (14)	3 (12)	
Diabetes mellitus, n (%)	3 (21)	14 (56)	0.04
Hypertension, n (%)	11 (79)	22 (88)	0.65
Cardiovascular disease, n (%)	1 (7)	3 (12)	0.63
Time on peritoneal dialysis (months)	8 (6–20)	14 (8–48)	0.08
Urine output (mL)	70 (0–1000)	500 (0–700)	0.98
Systolic blood pressure (mmHg)	137 ± 25	133 ± 21	0.64
Diastolic blood pressure (mmHg)	89 ± 18	82 ± 14	0.22
Body mass index (kg/m^2^)	25.9 ± 4.0	27.0 ± 3.6	0.39
Constipation, n (%)	1 (7)	12 (48)	0.01
Gastrointestinal symptoms (score)	10 ± 2.1	12 ± 3.7	0.18
Protein energy wasting, n (%)	9 (64)	15 (60)	0.79
Low muscle mass *, n (%)	2 (14)	8 (32)	0.22
Energy intake (kcal)	1102 ± 355	1221 ± 397	0.36
Protein intake (g)	55 ± 28	57 ± 20	0.86
Fiber intake (g)	18 ± 6	18 ± 8	0.91

* Mid-arm muscle area below 10th percentile for age and sex.

**Table 2:** Comparison of biochemical and dialysis adequacy results according to the presence of cognitive impairment.

Variable	Normal Cognitive Function (n = 14)	Mild Cognitive Impairment (n = 25)	*p*
Dialysis volume (L/day)	10 (9.7–10.7)	10 (9.6–10)	0.77
Ultrafiltration (mL/day)	818 (534–1800)	862 (371–1153)	0.55
Total Kt/Vurea (L/week)	1.75 ± 0.59	1.89 ± 0.42	0.38
Residual kidney function (mL/min)	0.08 (0–3.1)	1.3 (0–3.0)	0.61
nPNA (g/kg)	0.82 ± 0.20	0.81 ± 0.13	0.89
Hemoglobin (g/dL)	11.3 ± 2.6	11.6 ± 2.44	0.73
Glucose (mg/dL)	98 ± 16	116 ± 48	0.10
Urea (mg/dL)	126 ± 31	120 ± 32	0.56
Creatinine (mg/dL)	14.9 ± 5.4	11.3 ± 3.7	0.02
Phosphorus (mg/dL)	5.9 ± 1.7	5.2 ± 1.2	0.15
Calcium (mg/dL)	8.3 ± 1.4	8.8 ± 0.7	0.40
Potassium (mmol/L)	4.6 ± 0.3	4.5 ± 0.6	0.58
Sodium (mmol/L)	141 ± 2.7	140 ± 3.1	0.19
Total cholesterol (mg/dL)	169 ± 53	176 ± 34	0.60
Triglycerides (mg/dL)	145 ± 95	132 ± 83	0.66
Albumin (g/dL)	4.03 ± 0.49	3.89 ± 0.41	0.38
C-reactive protein (mg/L)	0.85 (0.57–4.8)	1.7 (0.50–7.3)	0.50
Lipopolysaccharides (ng/mL)	58 (44–74)	49 (33–87)	0.53

nPNA: normalized protein nitrogen appearance.

## Data Availability

The raw data supporting the conclusions of this article will be made available by the authors on request.
